# Insomnia in Type 2 Diabetes Mellitus: Prevalence, Psychological Correlates, and Association With Glycemic Control

**DOI:** 10.7759/cureus.106629

**Published:** 2026-04-08

**Authors:** Carlos Siopa, Ana Duarte, João Revez Lopes, Diogo Telles Correia, Filipa Novais

**Affiliations:** 1 Psychiatry, Unidade Local de Saúde (ULS) de Santa Maria, Lisbon, PRT

**Keywords:** #insomnia, mental well-being, metabolic control, sleep pattern, type 2 diabetes mellitus

## Abstract

Purpose

This study aimed to assess the prevalence of insomnia in adults with type 2 diabetes mellitus and its association with glycated hemoglobin.

Methods

In this cross-sectional study, patients attending a diabetes clinic completed the Insomnia Severity Index and the Hospital Anxiety and Depression Scale (HADS). Glycated hemoglobin values were self-reported by participants based on their most recent laboratory result. Logistic and linear regression analyses evaluated demographic and psychological predictors of insomnia and the relationship between insomnia and glycated hemoglobin.

Results

The prevalence of clinically significant insomnia symptoms was 37.7%. Higher HADS total scores were independently associated with insomnia, while age, sex, and glycated hemoglobin were not significant predictors. Insomnia status was not significantly associated with glycated hemoglobin levels (coef.=0.04; p=0.28). However, age, sex, and glycated hemoglobin each independently predicted total HADS score (all p<0.01), suggesting a multifaceted interplay among demographic, metabolic, and psychological factors.

Conclusions

Insomnia affects over a third of adults with type 2 diabetes mellitus, but was not independently associated with glycated hemoglobin. Routine sleep and mental health screening, especially in younger patients, could improve well‑being and type 2 diabetes mellitus management.

## Introduction

Type 2 diabetes mellitus (T2DM) is a major global health challenge, contributing substantially to morbidity, mortality, and healthcare costs. Achieving adequate glycemic control, commonly assessed using glycated hemoglobin (HbA1c), is central to preventing microvascular and macrovascular complications. While factors such as diet, physical activity, and medication adherence are well established, emerging evidence highlights the previously underestimated role of sleep, particularly insomnia, in the development and management of T2DM [1]. Among sleep disorders, insomnia, defined in the Diagnostic and Statistical Manual of Mental Disorders, Fifth Edition (DSM-5), as persistent difficulty initiating or maintaining sleep or early morning awakening occurring at least three times per week for a minimum of three months, has emerged as a condition of relevance in individuals with T2DM [1].

In the general population, its prevalence ranges between 6% and 20%. However, among individuals with T2DM, reported rates vary widely, from 6% to as high as 80% [2,3]. For example, a large cohort study identified mild and severe insomnia in 23% and 10.7% of T2DM patients, respectively [4]. This heterogeneity likely reflects methodological differences, including the use of self-reported sleep measures, variability in clinical characteristics of the samples, and inconsistent control for psychiatric comorbidities.

On the one hand, patients with poor glycemic control, particularly those with elevated HbA1c, often report more frequent and severe insomnia symptoms [5]. A recent meta-analysis demonstrated that T2DM patients with insomnia had, on average, 0.23% higher HbA1c levels and fasting glucose elevated by 0.4 mmol/L compared to those without insomnia, differences that may meaningfully affect clinical outcomes [2]. Conversely, insomnia itself appears to be a risk factor for developing T2DM. In a prospective cohort study, Hein et al. reported that 21.13% of participants with persistent sleep complaints developed T2DM over time [6]. The risk increased with the duration of sleep disturbances, and odds ratios for this association ranged from 1.52 to 2.98, emphasizing the importance of early intervention and tailored prevention strategies [2].

Consequently, the true prevalence of insomnia and its clinical correlates in patients with T2DM remain incompletely characterized. The relationship between insomnia and glycemic control is similarly unresolved. Several observational studies and meta-analyses suggest that insomnia or short sleep duration is associated with modest but clinically meaningful increases in HbA1c [7,8]. Furthermore, in patients on metformin monotherapy, variations in sleep patterns did not consistently correlate with glycemic control, highlighting the potential influence of pharmacological treatment on this relationship [9].

Experimental research does, however, support a plausible biological link. Sleep restriction and early awakening have been shown to elevate blood glucose levels and increase insulin resistance [3,10-13].

Observational studies suggest that the metabolic impact of insomnia may be more pronounced in men than in women [14]. Proposed mechanisms include neuroendocrine dysregulation, increased insulin resistance, nocturnal hypoglycemia, and behavioral pathways such as impaired self-care, reduced physical activity, and unhealthy dietary choices driven by sleep deprivation and fatigue. Experimental studies further support a causal link, demonstrating that sleep restriction can acutely elevate glucose levels and worsen insulin sensitivity [15,16].

Behavioral changes may also play a role. Insomnia has been associated with decreased engagement in self-care behaviors (e.g., healthy eating, physical activity), often due to impaired decision-making and increased fatigue [16,17]. Hormonal imbalances, particularly involving ghrelin and leptin, may contribute further: a study found that just two nights of restricted sleep (four hours) significantly increased the levels of these hormones, promoting hunger and reducing satiety [10,12,14,18,19]. Despite these biologically plausible pathways, empirical findings are inconsistent. Multiple studies have reported weak or null associations between insomnia and HbA1c after adjustment for confounders such as body mass index, physical activity, or medication use [9]. Sex- and age-related differences have also been reported but remain poorly understood. As a result, insomnia is not currently addressed in most diabetes management guidelines, reflecting ongoing uncertainty regarding its clinical significance.

Given the high prevalence of sleep complaints in diabetes clinics and the inconsistent evidence linking insomnia to glycemic control, further research using well-characterized clinical samples and standardized assessment tools is warranted. There is a need to clarify whether previously reported associations between insomnia and HbA1c persist after accounting for key demographic and psychological factors, such as age, sex, anxiety, and depression.

Accordingly, the present study had two primary objectives: (1) to estimate the prevalence of insomnia in adults with T2DM and identify its main demographic and psychological correlates and (2) to examine the association between insomnia severity and HbA1c levels after adjustment for these factors. Rather than assuming a uniform relationship, this study sought to test whether insomnia contributes independently to glycemic control or whether its clinical relevance in T2DM is more strongly linked to co-occurring psychological distress. By clarifying these relationships, the study aims to inform a more nuanced understanding of the role of sleep disturbances in diabetes management and support targeted screening strategies that integrate sleep and mental health assessment.

## Materials and methods

Study design and setting

This was a single-center, cross-sectional observational study conducted at the outpatient diabetes clinic of the Associação Protectora dos Diabéticos de Portugal (APDP) in Lisbon, Portugal. The study protocol received approval from the Ethics Committee of APDP (approval number: 004/2024) and was conducted in accordance with the Declaration of Helsinki and applicable Portuguese legislation. Data were collected anonymously through an online structured survey (Google Forms, Google LLC, Mountain View, CA, USA) distributed to clinic patients. The online modality was chosen to maximize reach and maintain participant anonymity while collecting validated self-report measures. No personally identifiable information was recorded. Data were stored securely and accessed only by the research team for analysis, in accordance with applicable data protection regulations. The recruitment period extended from January to June 2024.

Participants and eligibility criteria

Eligible participants were adults (≥18 years) with a clinician-confirmed diagnosis of T2DM who attended the APDP outpatient clinic during the recruitment period and who were able to read and understand Portuguese. Individuals were excluded from the study if they were unable to provide informed consent.

Sample size and flow

**Figure 1:**
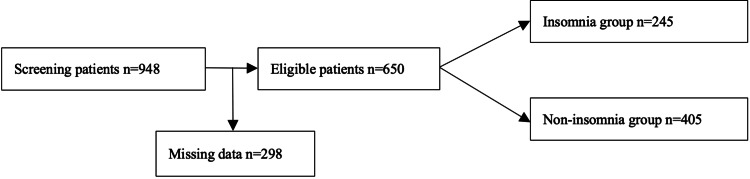
Flowchart of patient grouping.

Sample size was determined a priori to detect a small but clinically meaningful difference in HbA1c associated with insomnia, guided by prior meta-analytic estimates and assuming a 0.05 α and an 80% power. Allowing for incomplete responses, the target sample was set at ≈600. Of 948 patients screened, 298 were excluded for incomplete primary outcome data (missing Insomnia Severity Index (ISI) and/or HbA1c), yielding a final analytical sample of 650 (see Figure 1 for the flowchart).

Measures

The structured survey included the following components: The first was sociodemographic data, including age, sex, and educational attainment (measured in years of completed schooling). Secondly, participants were instructed to report their most recent HbA1c value; however, the exact date of measurement was not collected, and therefore the time interval between HbA1c assessment and questionnaire completion could not be determined. The third component was the ISI score, validated for the Portuguese population in assessing the severity of insomnia symptoms. The last was the Hospital Anxiety and Depression Scale (HADS) score, validated for the Portuguese population in assessing the presence of comorbid anxiety and depressive symptoms [20,21]. Formal permission to use both scales was obtained from the rights holder.

Insomnia symptoms were assessed using the ISI, a seven-item self-report questionnaire developed to evaluate the severity and impact of insomnia symptoms (Morin and Bastien et al.) and validated for European Portuguese, with demonstrated reliability and diagnostic accuracy [22-24]. ISI total scores (0-28) were analyzed both as a continuous measure and categorically. A cutoff of ≥14 was used to define clinically significant insomnia symptoms (screen-positive), based on validation studies for the European Portuguese population demonstrating good diagnostic accuracy [20].

Anxiety and depressive symptoms were assessed using the HADS, a Likert-type self-report instrument originally developed to screen for anxiety and depression in medical populations (Zigmond and Snaith) and validated for the Portuguese population (Pais-Ribeiro et al.) [22,25]. The scale comprises two subscales, anxiety and depression, each consisting of seven items rated on a four-point ordinal scale. Each item on the HADS is rated on a four-point ordinal scale, with response options ranging from 0 to 3. Higher scores indicate greater symptom severity. Specifically, the anxiety and depression subscales yield separate scores, with a total score classification as follows: 0-7 (normal), 8-10 (borderline), and 11-21 (clinical case). The internal consistency is high for both the anxiety and depression subscales (α=0.81 and α=0.78, respectively) [21,25]. The HADS was included to control for potential psychiatric comorbidities, since anxiety and depressive symptoms are common in T2DM and may confound the relationship between insomnia and glycemic control. By accounting for these factors, the study aimed to provide a more accurate assessment of the specific association between insomnia and HbA1c.

Data management and missing data

Data were exported from Google Forms into Stata (StataCorp LLC, College Station, TX, USA) for analysis. Responses with missing primary exposure (ISI) or outcome (HbA1c) were excluded from the primary analysis. The proportion of missing data was quantified for key variables, and complete-case analysis was used. No imputation procedures were applied due to the cross-sectional design and the nature of missingness. The mechanism of missingness could not be formally tested; therefore, the assumption of missing completely at random (MCAR) cannot be verified.

Statistical analysis

Statistical analyses were performed using Stata (version 14.2). Descriptive statistics were presented as mean±standard deviation. To test our hypothesis, three regression analyses were conducted with predefined outcomes. Binary logistic regression was used to identify correlates of clinically significant insomnia (ISI ≥14), with age, sex, HbA1c, and HADS total score entered as predictors. Second, as the primary analysis, linear regression examined the association between insomnia and glycemic control, with HbA1c (%) as the outcome and insomnia entered as the primary predictor (ISI total score and insomnia status), adjusting for age, sex, and HADS total score. Third, an exploratory linear regression examined psychological distress, with HADS total score as the outcome and age, sex, HbA1c, and ISI total score as predictors. Results are reported as odds ratios or regression coefficients with 95% confidence intervals. Multicollinearity between predictors was assessed using variance inflation factors (VIF), with all values below commonly accepted thresholds, indicating no problematic collinearity. All tests were two-sided, and p-values of ≤0.05 were considered statistically significant. Due to the nature of the dataset, information on several clinically relevant variables (e.g., body mass index, duration of diabetes, medication use, sleep apnea, and physical activity) was not available and therefore could not be included in the models.

## Results

Sample characteristics 

A total of 948 participants were screened, with ages between 26 and 94 years old and an average of 64 years, of whom 589 were females and 359 were males (62% and 38%, respectively). Of these, 298 (31.4%) were excluded due to missing primary exposure (ISI) and/or outcome (HbA1c) data, yielding a final analytical sample of 650 participants (response rate: 67.4%). Missingness in secondary variables was low and did not materially affect the analytical sample; therefore, complete-case analysis was considered appropriate. Educational attainment ranged widely, with the modal category corresponding to completion of secondary education. Participants included in the analytical sample were compared with those excluded due to missing ISI and/or HbA1c data. No significant differences were observed in age or sex between groups; however, educational level differed between included and excluded participants (see Appendices). Table 1 presents the characteristics of the final analytical sample (n=650).

**Table 1:** Demographic characteristics of the study population.

Demographic variables	n (%) or mean±SD
Age (years)	63.1±10.1
Sex	
Male	417 (64.2%)
Female	233 (35.8%)
Education level	
Lower education (≤3rd cycle)	61 (9.4%)
Secondary education	238 (36.6%)
Bachelor's degree	272 (41.9%)
Master's degree	60 (9.2%)
Doctoral degree	19 (2.9%)

Prevalence of insomnia

Among the 650 participants included in the analysis, 245 met the criteria for clinically significant insomnia symptoms (ISI ≥14), corresponding to a prevalence of 37.7%.

Predictors of insomnia (ISI ≥14)

A multivariable logistic regression analysis was performed to identify independent predictors of insomnia. No evidence of problematic multicollinearity was identified among model predictors. In the adjusted model, only the HADS total score was independently associated with insomnia. Higher HADS scores were associated with increased odds of insomnia (OR=1.19; 95% CI: 1.16-1.23; p<0.001). Age, sex, and HbA1c were not independently associated with insomnia (Table 2).

**Table 2:** Multivariable logistic regression for insomnia. HbA1c: glycated hemoglobin; HADS: Hospital Anxiety and Depression Scale

Predictor variable	OR	95% CI	P-value
Age	0.99	0.97-1.01	0.185
Sex	0.78	0.55-1.12	0.174
HbA1c	1.00	0.999-1.004	0.254
HADS total	1.19	1.16-1.23	<0.001

Sleep impairment as a predictor of HbA1c

Insomnia severity showed no meaningful relationship with HbA1c (coef.=0.011 per ISI point; 95% CI: -0.011 to 0.033; p=0.308). Likewise, age, sex, and psychological distress (HADS total) failed to predict glycemic levels (all p>0.20). These findings indicate that, after adjustment for demographic and psychological factors, sleep impairment did not independently predict HbA1c levels in this sample (Table 3). These models should be interpreted as minimally adjusted, as key clinical confounders were not available for inclusion.

**Table 3:** Multivariate linear regression analysis of predictors of HbA1c. ISI: Insomnia Severity Index; HbA1c: glycated hemoglobin; HADS: Hospital Anxiety and Depression Scale

Predictor variable	Coef.	95% CI	P-value
ISI	0.011	-0.011 to 0.033	0.308
Sex	0.056	-0.173 to 0.284	0.633
Age	0.002	-0.009 to 0.013	0.685
HADS	0.011	-0.006 to 0.028	0.209

Predictors of anxiety and depression

A higher ISI total score was strongly associated with greater psychological distress (p<0.001). Female sex and higher HbA1c were also independently associated with higher HADS scores, while increasing age was associated with lower HADS scores (all p<0.05) (Table 4).

**Table 4:** Linear regression analysis of predictors of HADS total score. HbA1c: glycated hemoglobin; HADS: Hospital Anxiety and Depression Scale

Predictor variable	Coef.	95% CI	P-value
Age	-0.17	-0.23 to 0.11	<0.001
Sex	-2.6	-3.80 to 1.38	<0.001
HbA1c	0.45	0.03 to 0.87	0.034

## Discussion

This study provides three main findings. Clinically significant insomnia symptoms were common (37.7%) and strongly associated with psychological distress. Insomnia severity was not associated with HbA1c after adjustment for other variables. The use of a validated instrument (ISI) and a clinically relevant cutoff (≥14) strengthen the comparability of our findings with existing literature. Importantly, insomnia was defined using a validated screening instrument rather than a formal clinical diagnosis, and results should be interpreted accordingly. Also, the regression models were adjusted for demographic and psychological variables but did not include other clinical variables such as body mass index, duration of diabetes, medication use, or sleep-related comorbidities. Therefore, residual confounding cannot be excluded, and the absence of an association between insomnia and HbA1c should be interpreted with caution.

Our findings are consistent with previous studies that did not demonstrate a statistically significant association between insomnia and metabolic control after adjustment for confounders (e.g., body mass index, physical activity, psychiatric symptoms) [9]. Although biological mechanisms linking sleep disturbance to glucose metabolism are established, these effects may be modest in real-world clinical populations and attenuated by routine diabetes care.

Psychological distress (HADS total score) was the only independent predictor of insomnia in the adjusted model. Each incremental increase in HADS score was associated with a substantial increase in the odds of insomnia, underscoring the central role of affective symptoms in sleep disturbances within this population. Given the conceptual overlap between insomnia symptoms and psychological distress, the absence of problematic multicollinearity in our models supports the robustness of the observed associations. This finding highlights a strong and clinically meaningful link between insomnia and emotional distress in individuals with T2DM. These results are in line with previous studies demonstrating bidirectional relationships between insomnia and mood disorders [26]. Several factors may explain the absence of an independent association, including measurement limitations (self-reported HbA1c and timing variability), heterogeneity of insomnia, and attenuation of metabolic effects by routine diabetes treatment [27].

Two secondary findings deserve emphasis: insomnia prevalence in this clinic sample (37.7%) is substantial and clinically relevant and the association between insomnia and age observed in unadjusted analyses was not maintained in the multivariable model. This suggests that apparent age-related effects may reflect underlying differences in psychological distress rather than a direct relationship with sleep disturbance. Such findings emphasize the importance of adequately adjusting for mental health variables when studying sleep in chronic medical conditions. Additionally, higher HbA1c was independently associated with greater psychological distress in our models. These findings highlight a complex interplay between sleep, mood, and metabolic status. Clinically, these findings support prioritizing routine screening for psychological distress and sleep disturbances in patients with T2DM.

Strengths and limitations

This study includes a large clinic-based sample (n=650), use of validated instruments for insomnia (ISI) and anxiety/depression (HADS), and prespecified analytic models that explicitly separated the main question from correlative analyses. However, a cross-sectional design precludes temporal or causal conclusions; HbA1c values were self-reported and may not have been temporally aligned with the insomnia assessment. Given that HbA1c reflects glycemic control over the preceding 2-3 months, variability in the timing of measurement may have attenuated potential associations and contributed to null findings. These findings should be interpreted considering the use of self-reported HbA1c and the absence of standardized timing, which may have reduced measurement precision. A substantial proportion of participants were excluded due to missing primary data (31.4%), which may introduce selection bias and limit generalizability. Although comparisons between included and excluded participants did not reveal differences in age or sex, the observed differences in educational level suggest that missingness may be related to socioeconomic factors, further supporting the possibility of non-random missingness. Additionally, the lack of data on other clinical variables (e.g., body mass index, diabetes duration, medication use, and sleep comorbidities) limited the ability to fully adjust for potential confounding factors. As such, the analyses should be considered minimally adjusted, and residual confounding may have influenced the observed associations.

Because the mechanism of missingness could not be established, it is possible that missing data were not completely at random. If insomnia severity, psychological distress, or metabolic control was associated with non-response, the observed estimates may be biased.

Future directions and implications

Future work should prioritize prospective designs and objective sleep measurement (actigraphy or polysomnography) to delineate temporal sequences and phenotype-specific effects. Randomized trials of insomnia treatment (e.g., cognitive behavioral therapy for insomnia (CBT-I)) with glycemic endpoints would determine whether improving sleep can meaningfully influence metabolic control. Clinically, our findings support routine screening for sleep disturbance and psychological distress in diabetes clinics, with integrated pathways for mental health assessment and treatment.

## Conclusions

In this clinic sample of adults with T2DM, insomnia symptoms were highly prevalent and strongly associated with psychological distress, but were not independently associated with HbA1c after multivariable adjustment. These results suggest that, within routine clinical populations, insomnia's most immediate impact may be on emotional well-being, therefore highlighting the importance of routine screening for insomnia and psychological distress in diabetes care and underscoring the need for longitudinal and interventional studies to clarify the potential metabolic impact of sleep disturbances.

## Appendices

**Table 5:** Comparison between included and excluded participants. This table compares participants included or excluded in the analytical sample due to missing data (n=298), across available demographic variables. Continuous variables are reported as mean±standard deviation and categorical variables as number (percentage).

Variables	Included (n=650)	Excluded (n=298)	P-value
Age (years)	63.1±10.1	64.2±10.4	0.129
Sex			
Male	417 (64.2%)	178 (59.7%)	0.240
Female	233 (35.8%)	120 (40.3%)	
Education level			
Lower education (≤3rd cycle)	61 (9.4%)	38 (12.8%)	0.007
Secondary education	238 (36.6%)	107 (35.9%)	
Bachelor's degree	272 (41.9%)	117 (39.3%)	
Master's degree	60 (9.2%)	28 (9.4%)	
Doctoral degree	19 (2.9%)	8 (2.6%)	
